# Chemical composition of some seaweed from Mediterranean Sea coast, Egypt

**DOI:** 10.1007/s10661-012-3009-y

**Published:** 2012-12-05

**Authors:** Ghada F. El-Said, Amany El-Sikaily

**Affiliations:** Environmental Division, National Institute of Oceanography and Fisheries, Alexandria, Egypt

**Keywords:** Marine seaweeds, Some elements, Carbohydrate, Ion quotient, Hazard index, Mediterranean Sea coast, Egypt

## Abstract

This study pointed to the assessment of the chemical composition (F, Ca, Mg, Na, K, Fe, Mn, Zn, Cu, Ni, Co, Cr, Cd, and carbohydrate) of different marine seaweeds (red, green, and brown) from the Egyptian Mediterranean Sea coast. The results showed that green seaweeds supplied better calcium sources than the red and brown ones. Also, red and brown seaweeds showed higher averages of Na and K than that in green species and these seaweeds could play an important role in the electrolyte balance in humans. On the other hand, green seaweeds gave the highest average carbohydrate concentration; thus, these green species could be used as a source of polysaccharides. Ion quotient values for almost seaweed species were between 1.4 and 4.0, so they can reduce hypertension, preeclampsia, and heart disease in human beings. Interestingly, the calculated hazard quotient of elements was below 1. Accordingly, these seaweed species were of high quality and safety and might be used in the field of nutrition.

## Introduction

Marine seaweeds comprise few thousand of species and they represent a considerable part of the littoral biomass. According to their nutrient value and chemical composition, they are classified as red (Rhodophyta), brown (Phaeophyta), and green seaweeds (Chlorophyta) (Dawczynski et al. 2007). Many seaweed species are used in the industry, principally for the extraction of phycocolloids (Jimenez-Escrig and Sanchez-Muniz 2000) and as a source of pharmaceutical substances. Also, they are used as herbal medicine, fertilizer, fungicides, and herbicides and for the direct use in human nutrition, too (Ortiz et al. 2006; Aguilera-Morales et al. 2005; Cardozo et al. 2007). Seaweeds are known as a highly nutritive food containing vitamin, protein, mineral, fiber contents, and essential fatty acids (Ortiz et al. 2006). They were traditionally used in Chinese, Japanese, and Korean diet since ancient times (Dawczynski et al. 2007). Additionally, they can be eaten in raw salads, soups, cookies, meals, and condiments (Aguilera-Morales et al. 2005). Specially, red and brown seaweeds are mainly used as human food sources. Nowadays, seaweeds consumption is increasing due to their natural composition. However, they contain 80–90 % water and their dry weight basis contains 50 % carbohydrates, 1–3 % lipids, and 7–38 % minerals. Their protein contents are highly variable (10–47 %) with high proportions of essential amino acids (García-Casal et al. 2007). Because of their low fat abundance and the presence of protein and carbohydrate substances, they can contribute few calories to the diet (Rupérez 2002). However, the chemical composition and the abundance of carbohydrates vary among seaweed species. Red seaweeds varieties consist of different typical carbohydrates kinds including: floridean starch (α-1,4-bindingglucan), cellulose, xylan, and mannan. Moreover, their water-soluble fiber fraction is formed by sulfur-containing galactans, e.g., agar and carrageenan (Jimenez-Escrig and Sanchez-Muniz 2000). On the other hand, the typical carbohydrates in brown seaweeds varieties consist of fucoidan, laminaran (β-1,3-glucan), cellulose, alginates, and mannitol. Brown seaweeds, fibers are mainly cellulose and insoluble alginates. These alginates are Ca, Mg, or Na salts of alginic acid (1,4-linked polymer of β-d-mannuronic acid and α-l-guluronic acid). In contrast, the amorphous, slimy fraction of fibers consists mainly of water-soluble alginates and/or fucoidan. The main reserve polysaccharides of Phaeophyta are laminaran (β-1,3-glucan) and mannitol (Kolb et al. 1999). The typical seaweeds’ carbohydrates are not digestible by the human gastrointestinal tract and, therefore, they are dietary fiber. The content of total dietary fiber ranges from 33–50 g/100 g d.w. (Lahaye 1991; Jimenez-Escrig and Cambrodon 1999; Ruperez and Saura-Calixto 2001). For example, Japanese people consume more than 1.6 kg seaweed dry weight per year per capita (Fleurence 1999). Moreover, because of their minerals presence (Na, K, Ca, Mg, Fe, Zn, Mn, etc.) they are needed for human nutrition. However, this wide range in mineral content (8–40 %) is not found in edible land plants, due to many factors such as; seaweed phylum, geographical origin, and seasonal, environmental and physiological variations (Nisizawa et al. 1987; Rupérez 2002). Seaweeds are also one of the most important vegetable sources of calcium. Their calcium content may be as high as 7 % of the dry weight and up to 25 to 34 % in the chalky seaweed, lithothamnion. So, seaweed consumption may be useful in the case of expectant mothers, adolescents, and elderly that all exposed to a risk of calcium deficiency (Burtin 2003). Additionally, Egyptian seaweeds contain considerable concentrations of fluoride of 19.17–53.70 mg/g (Masoud et al. 2006). Fluoride (F) is considered as an essential element; primarily because of its benefits to dental health and its suggested role in maintaining the integrity of bone (ATSDR (Public Health Service, Agency for Toxic Substances and Disease Registry) 2003). Moreover, physiologically active of the extracted substances from various seaweeds have been studied (Murata and Nakazoe 2001). Seaweeds appear to be an interesting source for ethnomedicinal and phytochemical studies. On the one hand, the power of algal resources has been sought for thousands of years for their ability to prevent disease and prolong life. However, they have shown high potential in controlling antimicrobial, antitumor, anticoagulant, and cytotoxic activity (Sabina et al. 2005). In Egypt, seaweeds were exposed to few studies for evaluating their human nutrition importance (Abdallah 2007; Abdallah 2008). These studies improve their recommendation for human consumption. However, *Enteromorpha compressa* had 6.6–11.3 % protein, 4.1–4.2 % total lipids and 8.7–8.16 % carbohydrate. Also, they contained a total concentration of Na, K, Ca and Mg ranging from 2,689 to 4,840 mg/100 g and total content of Fe, Zn, Mn, and Cu was 118–199 mg/100 g, more than those reported for edible land plants (Abdallah 2007). Furthermore, *Padina pavonia* (brown seaweed), *Pterocladia capillacea* (red seaweed), and *Ulva lactuca* (green seaweed) showed protein and lipid contents of 143.7 and 126.9 mg/g and 5.7 and 8.0 %, respectively, and total element concentrations for Na, K, Ca, and Mg, and Fe, Zn, Mn, and Cu of 18,291–26,178 and 35.1–125.5, respectively (Abdallah 2008).

In view of the current increasing demand for seaweed products, the aim of this work was to study the nutritional value for some seaweed (red, green, and brown) from Mediterranean Sea, Alexandria coast, Egypt, in order to assess their validity towards the human consumption.

## Materials and methods

### Area of study

**Fig. 1:**
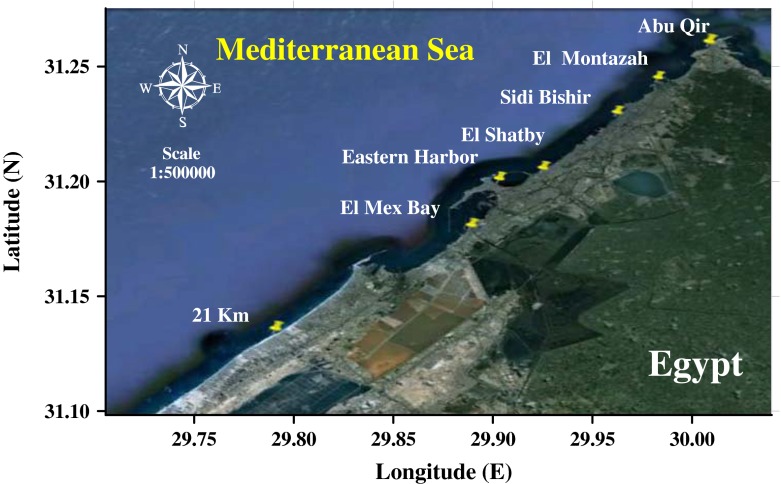
Sampling locations along Alexandria Mediterranean Sea coast, Egypt

Different seaweed species of different classes including, red (*Jania rubens*, *Gracilaria compressa*, *Gracilaria verrucosa*, *Pterocladia capillacea*, and *Hypnea musciformis*), green (*Ulva lactuca*, *Codium tomentosum*, and *Enteromorpha intestinalis*), and brown ones (*Colpomenia sinuosa* and *Sargassum linifolium*) were collected from seven locations (Abu Qir Bay, El Montazah, Sidi Bishir, El Shatby, Eastern Harbor, El Mex Bay, and 21 km) along Egyptian Mediterranean coast (Alexandria) during April 2011 (Fig. 1). These locations were selected to cover the expected polluted areas (Abu Qir Bay, Eastern Harbor and El Mex Bay) including industrial and/or human activities as well as unpolluted regions (El Montazah, Sidi Bishir, El Shatby, and 21 km). The area of study was extended from 31.318991° N, 30.058157° E to 31.096231° N 29.726527° E. However, Abu Qir Bay lies along the Egyptian Mediterranean coast of about 25 km east of Alexandria. This bay is semicircular with an average water depth of 10 m. It is bordered by Abu Qir headland and Rosetta headlands in its eastern side. The rocks of Abu Qir Bay have numerous small and fine holes that afford excellent domains for seaweed attachment. So, many seaweed species are found along this bay. This area is affected by brackish water coming through El Maadya which connects to Abu Qir Bay and Lake Edku. This bay receives huge amounts of sewage, agricultural, and industrial discharged wastes from El Tapia pumping station; while El Montazah, Sidi Bishir, El Shatby, and 21 km are affected by the water current from the west to the east. Eastern Harbor location is a semicircular basin with an area of 2.53 km^2^ and an average depth of 6 m. It is one of the main fishing and hatching harbors of Alexandria. El Mex Bay location is laying at the western side of Alexandria with a maximum depth of 20 m. It receives huge amounts of untreated agriculture, industrial, as well as sewage discharged waters from El Umum drain. Seawater temperature and pH were fluctuated from 21.0 to 23.0 °C and 7.6 to 8.2, respectively.

### Sampling

The seaweeds’ samples were picked with hand and immediately washed with seawater to remove the foreign particles, sand particles and epiphytes. Then it was kept in an ice box and immediately transported to the laboratory and washed thoroughly with tap water to remove the salt on the surface of the sample. After that, the samples were identified by species (Riedel 1970; Aleem 1993). They were spread on blotting paper to remove excess water. The dry air samples were placed in an oven at 50 °C and water content was calculated. Pulverized in the grinder and sieved through a screen with an aperture of 0.5 mm. Then, the powdered material was kept in airtight plastic bottles at room temperature until analysis.

### Chemical analyses

#### Elemental analysis

For all studied elements analysis except fluoride, 0.20 g of dried fine-powdered seaweeds was completely digested in a well-stoppered Teflon cup using a mixture of concentrated HNO_3_, HClO_4_, and HF acids (3:2:1 *v*/*v*, Merck (Germany)). However, each sample was triplicate digested. All the Teflon cups were placed inside a well-closed stainless steel block, which achieve high pressure, and heated by a controlled thermostatic plate at 50 °C until digestion performance. Each final solution was diluted to 25 ml with distilled deionized water into a polytetrafluoroethylene flask and then filtered into an acid-clean PVC bottle. All digested solutions were analyzed in triplicate using Perkin Elmer 2830 flame Atomic Absorption Spectrophotometer for metals determination. On the other hand, sodium and potassium in the same digested samples was determined by means of Corning Clinical flame photometer 410C. A blank was prepared using the same procedure. Normal precautions for element analysis were performed throughout these steps. However, all the glassware and the Teflon cups were previously soaked overnight with 20 % HNO_3_ and then rinsed by it. Each element concentration was estimated quantitatively according to the standard conditions described in the instrument manual. However, working standards of studied elements were prepared by diluting concentrated stock solutions with deionized water (Merck, Germany). Reagents of analytical grade were utilized for the blanks and calibration curves; precision was checked against standard reference material (IAEA-433, International Atomic Energy Agency; Analytical Quality Control Services) which was analyzed with the digested seaweed solutions during the analysis course. The measured concentrations of heavy metals and sodium, potassium and magnesium were within the range of certified values with a recovery of 96.1–106.1 %, whereas precision was agreed to be within 10 % (Table 1). The recoveries of studied metals were 105.5 % for Cd; 96.9 % for Co; 69.7 % for Cu; 105.2 % for Cr; 101.4 % for Mn; 99.6 % for Ni; 98.0 % for Zn; 96.1 % for Fe; 103.6 % K; 96.3 % for Na and 106.1 % for Mg. The absorption wavelength and detection limits of heavy metals were as follows: 228.8 nm and 0.006 μg/g for Cd; 240.7 nm and 0.009 μg/g for Co; 324.7 nm and 0.008 μg/g for Cu; 279.5 nm and 0.006 μg/g for Mn; 232.0 nm and 0.009 μg/g for Ni; 213.9 nm and 0.004 μg/g for Zn and 248.3 nm and 0.007 μg/g for Fe, respectively.

**Table 1:** Average and standard deviation obtained for four replicates of the standard reference material IAEA-433

Metal	Certified	Measured	Recovery%
Cadmium^a^	0.531 ± 0.033	0.161 ± 0.037	105.49
Cobalt^a^	12.9 ± 1.2	12.5 ± 1.5	96.90
Copper^a^	30.8 ± 2.6	29.9 ± 3.0	96.73
Chromium^a^	136.0 ± 10.0	134.0 ± 11.0	105.15
Manganese^a^	316.0 ± 16.0	321.0 ± 20.0	101.43
Nickel^a^	39.4 ± 3.1	39.2 ± 3.5	99.56
Zinc^a^	101.0 ± 8.0	99.0 ± 3.5	98.02
Iron^b^	40.8 ± 1.9	39.2 ± 3.2	96.10
Potassium^b^	16.6 ± 3.2	17.2 ± 1.3	103.6
Sodium^b^	13.5 ± 1.5	13.0 ± 0.9	96.30
Magnesium^b^	11.5 ± 0.9	12.2 ± 0.7	106.10

^a^In milligrams per kilogram

^b^In grams per kilogram

#### Fluoride analysis

For fluoride digestion, each 0.2 g of dried fine-powdered seaweed’s samples were digested using 3 ml of a concentrated perchloric acid (Merck, Germany) in Teflon cup at room temperature. After, complete digestion the digested samples were diluted to 25 ml measuring flasks by double distilled water. The digested solutions were stored in clean dried stoppered polyethylene bottles until they used for fluoride analysis. Fluoride ion concentration was determined by the colorimetric procedure of zirconium alizarin red S (Courtenary and Rex 1951; Masoud et al. 2004) using UV/Visible single beam Spectronic 21 D Milton Roy spectrophotometer.

#### Carbohydrate analysis

The total carbohydrate content was assayed by the phenol/sulfuric acid method (Dubois et al. 1956) after extraction with 2.5 N HCl. The results were calculated from a glucose standard curve using UV/Visible single beam spectronic 21 D Milton Roy spectrophotometer (Schuep and Schierle 1995). Carbohydrate content was expressed as mg/g dry weight.

#### Statistical analyses

STATISTICA software, version 5, was used in the current study for the calculation of Pearson’s correlation coefficient matrix, estimating multiple regression as well as constructing a cluster analyses.

## Results and discussion

### Elemental analysis

Table 2 illustrates the average concentration of some elements in red, green and brown seaweed species. The present results showed the same elements ordering in seaweed species, except in brown seaweeds, iron (Fe) seems to be higher than zinc (Zn); F > Na > K > Ca > Mg > Zn > Fe > Mn > Co > Cd > Ni > Cu > Cr. Also, red seaweed species contain the highest iron (789.00 ± 40.02 μg/g) and zinc (1,088.67 ± 1,998.25 μg/g) average concentrations. In contrast, brown seaweeds have the lowest iron (40.26 ± 4.05 μg/g) and zinc (20.91 ± 1.36 μg/g) average ones. Whereas, it was stated that the elements content in seaweeds may be dependent on various environmental factors including; concentrations of elements in water (Andrade et al. 2004), interactions between elements, salinity, pH, light intensity, and metabolic factors such as dilution of element contents due to seaweed growth (Zbikowski et al. 2006). Also, concentrations of elements in seaweeds are regulated to a large extent by metabolic requirements (Zbikowski et al. 2006). Red, green, and brown seaweeds give Ca/Mg averages of 2.23 ± 0.98, 5.41 ± 4.10, and 2.89 ± 0.58, respectively (Table 2). Thus, green seaweeds supply better calcium sources than the red and brown ones. Accordingly, the high significant correlation between calcium and magnesium (*r* = 0.4969; *p* < 0.05) may be accompanied with the substitution of calcium by magnesium in calcite seaweed’s component. Also, green seaweeds show smaller average Na and K than red and brown seaweeds (Table 2). Whereas, sodium and potassium in the present data are strongly related (*r* = 0.4677; *p* < 0.05) as they play an important role in the electrolyte balance (Krishnaiah et al. 2008). It was mentioned that seaweeds living in ocean containing predominantly Na and their salts. Some seaweed accumulates more K and their salts than Na. However, potassium is an essential element for the growth and metabolic activities of plants and seaweeds (Sivakumar and Arunkumar 2009). The K/Na balance is regarded to be important for people who take diuretics, to control hypertension and suffer from excessive excretion of potassium (Cutler 2006; Zillich et al. 2006). Elements are also important as constituents of bones, teeth, soft tissues, hemoglobin, muscle, blood, and nerve cells, and are vital for overall mental and physical well being (Miyake et al. 2005; Kuda and Ikemori 2009).

**Table 2:** Distribution of some elements and carbohydrate contents in different seaweed species along the studied locations

Location	Seaweeds	Sample	Ca (mg/g)	Mg (mg/g)	Ca/Mg	Na (mg/g)	K (mg/g)	F (mg/g)	Carbohydrate (mg/g)
	Red seaweeds								
Abu Qir		*Jania rubens*	3.32	2.87	1.16	13.18	2.83	97.07	129.55
Abu Qir		*Gracilaria compressa*	3.33	1.73	1.92	29.08	4.46	40.97	116.23
Eastern Harbor		*Gracilaria verrucosa*	0.94	0.29	3.24	22.80	8.17	113.12	111.46
Abu Qir		*Pterocladia capillacea*	3.05	2.08	1.47	17.92	8.32	177.88	96.37
Abu Qir		*Pterocladia capillacea*	1.39	1.41	0.99	10.02	9.42	77.89	111.98
El Mex		*Pterocladia capillacea*	3.85	1.17	3.29	25.00	0.50	98.60	78.87
21 km		*Pterocladia capillacea*	10.49	4.24	2.47	39.66	8.37	164.06	80.64
Abu Qir		*Hypnea musciformis*	3.79	1.15	3.30	24.22	8.22	50.90	111.72
		Average	3.77	1.87	2.23	22.74	6.29	102.56	104.60
		SD	2.92	1.22	0.98	9.34	3.26	48.82	17.78
	Green seaweeds								
Abu Qir		*Ulva lactuca*	2.86	1.73	1.65	21.91	7.70	93.26	111.45
El Montaza		*Ulva lactuca*	6.47	0.56	11.55	6.37	1.68	85.03	109.49
Sidi Bishir		*Ulva lactuca*	1.89	1.14	1.66	21.69	7.23	89.20	115.39
El Shatby		*Ulva lactuca*	2.34	0.28	8.36	1.46	1.21	88.94	112.76
Eastern Harbor		*Ulva lactuca*	1.90	1.44	1.32	8.37	7.64	82.03	114.08
El Mex		*Ulva lactuca*	2.79	0.28	9.96	6.04	1.45	128.23	111.45
21 km		*Ulva lactuca*	16.73	2.54	6.59	3.33	4.43	109.03	101.62
Abu Qir		*Codium tomentosum*	5.50	0.83	6.63	11.79	4.29	100.17	111.45
Abu Qir		*Enteromorpha intestinalis*	2.77	2.81	0.99	22.12	8.65	101.89	87.20
		Average	4.81	1.29	5.41	11.45	4.92	97.53	108.32
		SD	4.75	0.93	4.10	8.36	2.99	14.40	8.84
	Brown seaweeds								
Abu Qir		*Colpomenia sinuosa*	3.77	1.14	3.31	24.51	9.19	144.74	118.00
Abu Qir		*Sargassum linifolium*	1.44	0.58	2.48	22.01	10.55	166.66	85.88
		Average	2.61	0.86	2.89	23.26	9.87	155.70	101.94
		SD	1.65	0.40	0.58	1.77	0.96	15.50	22.71

The average fluoride is ranged from 97.53 ± 14.40 to 155.70 ± 15.50 mg/g dry weight in green and brown seaweeds, respectively (Table 2). The high fluoride in brown seaweeds can explained by its significant correlation with potassium (*r* = 0.8028; *p* < 0.05). Thus, fluoride possibly increases the growth and metabolic activities of brown seaweeds (Camargo 2003). However, soluble fluorides are bioaccumulated by some aquatic and terrestrial biota (Liteplo et al. 2002). Fluorides can be taken up by aquatic organisms directly from the water or, to a lesser extent, via food. Their uptake is depending on the anthropogenic sources, the local geology and the physicochemical conditions (Liteplo et al. 2002; Camargo 2003). Fluoride has both positive and negative effects on human health. It is important to metabolism, formation and structure of bone and teeth, growth and reproduction and other physiological process in human body (Liteplo et al. 2002). Whereas long-term exposure or intake of fluoride can lead to fluorosis with symptoms such as changing in the bone structure and may also lead to enzyme inhibition. Oral exposure to fluoride may produce effects including nausea, vomiting, abdominal pain, diarrhea, fatigue, drowsiness, coma, convulsions, cardiac arrest, and even death. Calcification of muscles, osteosclerosis, and decreased production of erythrocytes may be also developed as a result of long-term exposure. (Liteplo et al. 2002; ATSDR 2003; Fawell et al. 2006; FSANZs 2008).

### Carbohydrate content

**Fig. 2:**
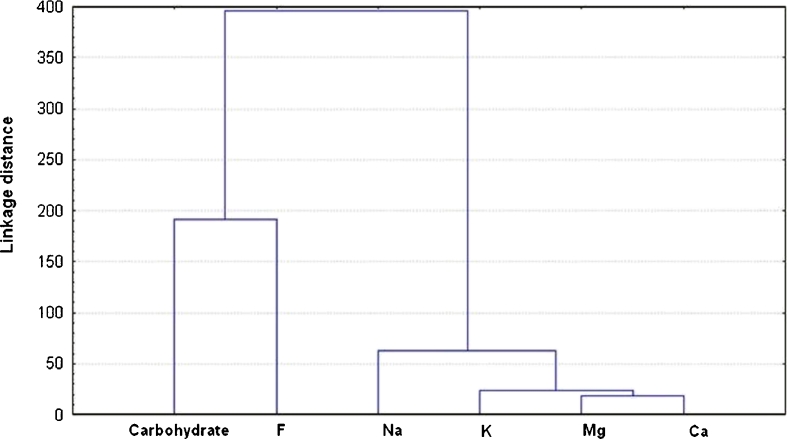
Cluster analysis for some elements and carbohydrate in different seaweeds

The total carbohydrates are found to be in considerable high values in the green seaweed species followed by red and then brown ones with average concentrations of 108.32, 104.6 and 101.94 mg/g, respectively (Table 2). Its occurrence is a function to the intensity of sunlight (El-Tawil and Khalil 1983). Red seaweed, *Pterocladia capillacea* (El Mex Bay), records the lowest concentration (78.87 mg/g) and the highest concentration is identified in *Jabia rubens* (129.55 mg/g). Meanwhile, its concentration in green species varies from 87.20 to 115.39 mg/g in *E. intestinalis* and *Ulva lactuca* from Abu Qir Bay and Sidi Bishir locations, respectively. Also, for brown seaweeds, the minimum concentration is recorded in *Sargassum linifolium* from Abu Qir Bay (85.38 mg/g) and the maximum content presents in Abu Qir in *Coupomenia simosa* (118 mg/g). These variations may attribute to species difference and to differences in their habitat, metabolic preferences. (Pádua et al. 2004). Accordingly, owing to our results the studied seaweeds can be used as a source for polysaccharides. On the other hand, correlation matrix analysis refers to a negative significant correlation between total carbohydrates and fluoride (*r* = −0.4912; *p* < 0.05). This relation is confirmed by the following multiple regression as well as cluster analyses (Fig. 2)

$$ \mathrm{F} = 227.81 + 0.058\ \mathrm{Ca} + 0.014\ \mathrm{Mg} + 0.15\ \mathrm{Na} + 0.311\ \mathrm{K}\text{-} 0.49\ \mathrm{Ca}\mathrm{rbohydrate} $$

Generally, from the point of view of the different used statistical analyses, fluoride seems to affect growth, metabolism, and other variable physiological processes in the seaweed species.

### Ion quotient

To characterize mineral waters, their hardness, calcium and magnesium contents, as well as the ratio of the latter are usually calculated. Thus, sodium and potassium contents are generally not taken into account. Because of Na contribution to high blood pressure ion quotient is used. It provides better dietary and sanitary characteristics than the simple Ca/Mg ratio. The ion quotient can be calculated for all living organisms (plants and animals, including mankind). It can be calculated by applying the following equation with concentrations given in moles (Kiss et al. 2004; Csikkel-Szolnoki et al. 2000).

$$ \mathrm{Ion}\ \mathrm{quotient} = \left( {\left[ {\mathrm{C}{{\mathrm{a}}^{+2 }}} \right] + \left[ {\mathrm{N}{{\mathrm{a}}^{+}}} \right]} \right)/\left( {\left[ {\mathrm{M}{{\mathrm{g}}^{+2 }}} \right] + \left[ {{{\mathrm{K}}^{+}}} \right]} \right) $$

Table 3 shows that the ion quotients for all seaweed species are between 1.4 and 4.0 except six ones, this mole ratio generally vary between 2.5 and 4.0 in human body. Accordingly, this means, that the feeding by the studied species can decrease the 2.5–4.0 ion quotient range in human body and reduce hypertension, preeclampsia, and heart disease.

**Table 3:** Ion quotients in the different seaweed species

Location	seaweeds	Algal species	$\mathrm{Ion}\ \mathrm{quotients}\left( {\left[ {\mathrm{Ca}{{}^{+2 }}} \right] + \left[ {\mathrm{N}{{\mathrm{a}}^{+}}} \right]/\left[ {\mathrm{Mg}{{}^{+2}}} \right] + \left[ {{{\mathrm{K}}^{+}}} \right]} \right)$
Abu Qir	Red seaweeds	*Jania rubens*	2.40
Abu Qir		*Gracilaria compressa*	5.58^a^
Eastern Harbor		*Gracilaria verrucosa*	4.46^a^
Abu Qir		*Pterocladia capillacea*	2.43
Abu Qir		*Pterocladia capillacea*	1.42
El Mex		*Pterocladia capillacea*	11.73^a^
21 km		*Pterocladia capillacea*	3.99
Abu Qir		*Hypnea musciformis*	4.08^a^
		Average	2.56
		SD	1.07
Abu Qir	Green seaweeds	*Ulva lactuca*	3.23
El Montazah		*Ulva lactuca*	6.74^a^
Sidi Bishir		*Ulva lactuca*	3.72
El Shatby		*Ulva lactuca*	3.34
Eastern Harbor		*Ulva lactuca*	1.46
El Mex		*Ulva lactuca*	6.69^a^
21 km		*Ulva lactuca*	3.04
Abu Qir		*Codium tomentosum*	4.42
Abu Qir		*Enteromorpha intestinalis*	2.43
		Average	3.09
		SD	0.94
Abu Qir	Brown seaweeds	*Colpomenia sinuosa*	3.81
Abu Qir		*Sargassum linifolium*	3.24
		Average	3.53
		SD	0.41

^a^Different data

### Human hazard index

The human hazard of the determined elements (Co, Mn, Zn, Cd, Cu, Cr, Ni, Fe, and F) in the studied seaweeds was studied to evaluate their nutritional value for humans (Albering et al. 1999; Port Angeles Harbor Sediment Characterization Study Port Angeles (2008)):

Ingestion of seaweeds (Albering et al. 1999):The ingestion of seaweeds is calculated by applying the following equation: $\mathrm{Ingestion}\ \mathrm{of}\ \mathrm{seaweed}\left( {\mathrm{mg}/\mathrm{kg}/\mathrm{day}} \right)=\frac{{\mathrm{CF}\times \mathrm{IRF}\times \mathrm{FI}\times \mathrm{AF}}}{\mathrm{BW}}$

Where CF = concentration of the contaminant in seaweed (in milligrams per kilogram fresh weight (fw)); IR = ingestion rate (kilogram fw per day; EPA 1998) [0.010 and 0.029 kg fw day^−1^ for child and adult, respectively]; FI = fraction contaminated (unitless) [0.5 for both child and adult]; AF = absorption factor (unitless) [1 for both child and adult]; and BW = body weight (in kilogram) [7 and 70 kg for a child and an adult, respectively].

**Fig. 3:**
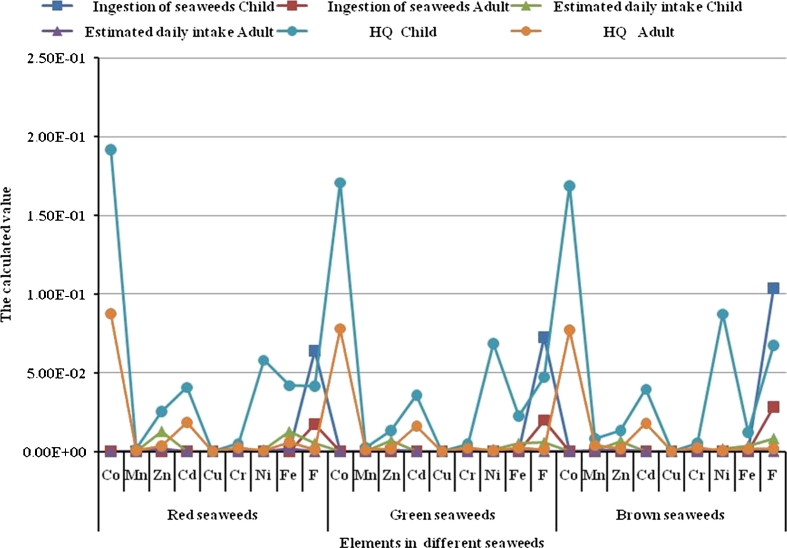
The calculated ingestion, estimated daily intake, and HQ values of some elements in the different seaweeds for child and adult

Generally, the ingestion of seaweeds values of all the determined elements for child are higher than those calculated for adult (Table 4 and Fig. 3). Among all the elements, fluoride shows the highest ingestion values of child and adult along the different seaweeds (6.40E-02, 7.27E-02 and 1.04E-01 and 1.75E-02, 1.99E-02, and 2.84E-02 mg/kg/day for red, green, and brown seaweeds, respectively).

**Table 4:** The calculated ingestion, estimated daily intake, and HQ values of trace elements in the different seaweeds for child and adult

Seaweeds	Element	Ingestion of seaweeds		Estimated daily intake		HQ	
		Child	Adult	Child	Adult	Child	Adult
Red seaweeds	Co	1.21E-05	3.31E-06	5.77E-05	2.64E-05	1.92E-01	8.79E-02
	Mn	3.44E-05	9.40E-06	1.64E-04	7.49E-05	1.64E-03	7.49E-04
	Zn	8.10E-04	2.22E-04	1.29E-02	1.77E-03	2.57E-02	3.53E-03
	Cd	8.58E-06	2.35E-06	4.09E-05	1.87E-05	4.09E-02	1.87E-02
	Cu	3.02E-06	8.26E-07	1.44E-05	6.58E-06	1.60E-04	7.31E-05
	Cr	1.06E-06	2.91E-07	5.08E-06	2.32E-06	5.08E-03	2.32E-03
	Ni	4.88E-06	1.34E-06	1.16E-03	1.06E-05	5.82E-02	5.32E-04
	Fe	7.91E-04	2.17E-04	1.26E-02	1.73E-03	4.19E-02	5.75E-03
	F	6.40E-02	1.75E-02	5.09E-03	1.40E-04	4.17E-02	1.14E-03
Green seaweeds	Co	1.08E-05	2.94E-06	5.13E-05	2.34E-05	1.71E-01	7.82E-02
	Mn	5.15E-05	1.41E-05	2.46E-04	1.12E-04	2.46E-03	1.12E-03
	Zn	4.27E-04	1.17E-04	6.78E-03	9.30E-04	1.36E-02	1.86E-03
	Cd	7.52E-06	2.06E-06	3.58E-05	1.64E-05	3.58E-02	1.64E-02
	Cu	6.00E-06	1.64E-06	2.86E-05	1.31E-05	3.18E-04	1.45E-04
	Cr	9.93E-07	2.72E-07	4.74E-06	2.17E-06	4.74E-03	2.17E-03
	Ni	5.76E-06	1.58E-06	1.37E-03	1.26E-05	6.86E-02	6.28E-04
	Fe	3.27E-04	8.95E-05	5.20E-03	7.13E-04	2.26E-02	2.38E-03
	F	7.27E-02	1.99E-02	5.78E-03	1.58E-04	4.73E-02	1.30E-03
Brown seaweeds	Co	1.06E-05	2.91E-06	5.07E-05	2.32E-05	1.69E-01	7.73E-02
	Mn	1.77E-04	4.84E-05	8.44E-04	3.86E-04	8.44E-03	3.86E-03
	Zn	4.21E-04	1.15E-04	6.70E-03	9.19E-04	1.34E-02	1.84E-03
	Cd	8.31E-06	2.28E-06	3.97E-05	1.81E-05	3.97E-02	1.81E-02
	Cu	4.36E-06	1.19E-06	2.08E-05	9.51E-06	2.31E-04	1.06E-04
	Cr	1.14E-06	3.13E-07	5.45E-06	2.49E-06	5.45E-03	2.49E-03
	Ni	7.34E-06	2.01E-06	1.75E-03	1.60E-05	8.75E-02	8.00E-04
	Fe	2.31E-04	6.32E-05	3.67E-03	5.04E-04	1.22E-02	1.68E-03
	F	1.04E-01	2.84E-02	8.26E-03	2.26E-04	6.77E-02	1.86E-03

### Estimated daily intake for noncancer health effects

The estimated daily intake for the studied elements (Co, Mn, Zn, Cd, Cu, Cr, Ni, Fe, and F) in their noncancerous health contents using the following equation was calculated (Health Consultation, Land Crab Evaluation, National Oceanographic Atmospheric Administration Data 2006; Herbicide Risk Assessment for the Aquatic Plant Management Final Supplemental Environmental Impact Statement 2001):

$$ \mathrm{Estimated}\ \mathrm{daily}\ \mathrm{intake}\left( {\mathrm{mg}/\mathrm{kg}/\mathrm{day}} \right)=\frac{{C\times \mathrm{IR}\times \mathrm{EF}\times \mathrm{ED}}}{{\mathrm{BW}\times \mathrm{AT}}} $$

where *C* = average concentration (in milligrams per kilogram) of the contaminant; IR is the ingestion rate [0.1135 kg/ day (4-oz meal) and 0.227 kg/day (8-oz meal) for child and adult, respectively]; EF = exposure frequency, or number of exposure events per year of exposure (365 days/year); ED = exposure duration, or the duration over which exposure occurs [6 and 70 years for child and adult, respectively (lifetime exposure)]; BW = body weight (16 and 70 kg for child/toddler and adult, respectively); AT = averaging time, or the period over which cumulative exposures are averaged (noncancer/lifetime = ED × 365 days/year).

Among all the studied seaweeds, red species show the highest Zn, Fe and F estimated daily intake values for child (1.29E-02, 1.26E-02, 5.09E-03 mg/kg/day, respectively; Table 4 and Fig. 3). Accordingly, red seaweeds can be considered as a reach source of these previously mentioned elements.

### Hazard quotient

The potential for adverse effects resulting from exposure to noncarcinogens will be assessed by comparing the estimated daily intake of the contaminant to its RfD, yielding an hazard quotient (HQ), as follows (Port Angeles Harbor Sediment Characterization Study Port Angeles (2008)):

$$ \mathrm{HQ}=\frac{{\mathrm{Estimated}\;\mathrm{daily}\;\mathrm{intake}}}{\mathrm{RfD}} $$

Where, HQ = hazard quotient (unit less); Estimated daily intake (in milligrams per kilogram per day); RfD = reference dose (in milligrams per kilogram per day). However, a HQ of 1.0 for any element is used to assess acceptable exposure and is utilized as a reference point. A HQ that is less than or equal to 1.0 indicates that the potential exposure is within the degree of exposure that is considered acceptable or “safe” (CanNorth (Canada North Environmental Services Limited Partnership) 2007). On the other hand, the HQ value that is greater than 1.0 suggests that the exposure exceeds the acceptable exposure limit. Interestingly, the present calculations for the detected elements in the different studied seaweeds have values less than 1.0, and can be considered safe for human nutrition (Table 4 and Fig. 3).

## Conclusions

According to the recent researches that always discover and explore seaweeds benefits, this study concerned with the validity of different seaweeds species collected from the shoreline of Egyptian Mediterranean Sea coast for the nutrition purpose. The elemental (Ca, Mg, Na, K, Fe, Mn, Zn, Cu, Ni, Co, Cr, and Cd) and carbohydrate composition in different seaweeds (red, green, and brown) were determined. The results showed a positive evaluation of the nutritional quality and safety for these seaweeds. The ion quotients for almost species were between 1.4 and 4.0. So, they could decrease the ion quotient range in human body. The hazard index of all the calculated elements was below 1 for the seaweeds. Also, the daily exposure of fluoride for child and adult was generally below both the adequate intake (in milligrams per day) for Australian and New Zealand populations. Accordingly, it is advisable to use these seaweed species in the applied nutritional field.
